# Supplementary material for: Aminergic and peptidergic modulation of insulin-producing cells in Drosophila
Source: eLife. 2025 Mar 10;13:RP99548. doi: 10.7554/eLife.99548 (PMC11893105; doi:10.7554/eLife.99548)
Supplement: Supplementary file 1. [file elife-99548-supp1.docx]

**Supplementary file 1A: Abbreviations used for receptors, neuropeptides, and biogenic amines.**

| **Abbreviation** | **Full name** |
| --- | --- |
| **Biogenic amine receptors** | |
| 5-HT1A | 5-Hydroxytryptamine receptor 1A |
| 5-HT1B | 5-Hydroxytryptamine receptor 1B |
| 5-HT2A | 5-Hydroxytryptamine receptor 2A |
| 5-HT2B | 5-Hydroxytryptamine receptor 2B |
| 5-HT7 | 5-Hydroxytryptamine receptor 7 |
| CG13579 | Orphan |
| Dop1R1 | Dopamine 1-like receptor 1 |
| Dop1R2 | Dopamine 1-like receptor 2 |
| Dop2R | Dopamine 2-like receptor |
| DopEcR | Dopamine Ecdysone receptor |
| Oamb | Octopamine receptor in mushroom bodies |
| Octalpha2R | α2-adrenergic-like octopamine receptor |
| Octbeta1R | Octopamine beta 1 receptor |
| Octbeta2R | Octopamine beta 2 receptor |
| Octbeta3R | Octopamine beta 3 receptor |
| Oct-TyR | Octopamine-Tyramine receptor |
| TyR | Tyramine receptor |
| **Neuropeptide receptors** | |
| AstA-R1 | Allatostatin A receptor 1 |
| AstA-R2 | Allatostatin A receptor 2 |
| CCAP-R | Crustacean cardioactive peptide receptor |
| CCHa2-R | CCHamide-2 receptor |
| CG10738 | Orphan |
| CNMaR | CNMamide receptor |
| Dh31-R | Diuretic hormone 31 receptor |
| Dh44-R1 | Diuretic hormone 44 receptor 1 |
| Dh44-R2 | Diuretic hormone 44 receptor 2 |
| FMRFaR | FMRFamide receptor |
| hec | hector |
| InR | Insulin-like receptor |
| Lkr | Leucokinin receptor |
| NPFR | Neuropeptide F receptor |
| Pdfr | Pigment-dispersing factor receptor |
| rk | rickets |
| RYa-R | RYamide receptor |
| SIFaR | SIFamide receptor |
| sNPF-R | short Neuropeptide F receptor |
| SPR | Sex peptide receptor |
| TkR86C | Tackykinin-like receptor at 86C |
| TrissinR | Trissin receptor |
| **Biogenic amines** | |
| 5-HT | 5-Hydroxytryptamine (serotonin) |
| DA | Dopamine |
| OA | Octopamine |
| Tyr | Tyramine |
| **Neuropeptides** | |
| AstA | Allatostatin-A |
| DH31 | Diuretic hormone 31 |
| LK | Leucokinin |
| MS | Myosuppressin |
| sNPF | short Neuropeptide F |
| TK | Tachykinin |

**Supplementary file 1B: G-protein prediction scores for selected receptors in IPCs.** From http://athina.biol.uoa.gr/bioinformatics/PRED-COUPLE2

| **Receptor** | **Isoform** | **G-protein prediction scores** |
| --- | --- | --- |
| AstA-R1 | NP_524700  NP_726877 | Gi/o - 0.92  Gi/o - 0.92 |
| AstA-R2 | NP_524544  NP_001247352  NP_001263042 | Gi/o - 0.83  Gi/o - 0.79  Gq/11 - 0.47  Gi/o - 0.83 |
| Lkr | NP_647968 | Gq/11 - 0.97 |
| MsR1 | NP_647713  NP_001261324 | Gi/o - 0.99  Gs - 0.32  Gi/o - 0.96 |
| MsR2 | NP_647711  NP_728735  NP_001261323 | Gi/o - 0.97  Gq/11 - 0.48  Gi/o - 0.97  Gq/11 - 0.48  Gi/o - 0.99 |
| TkR86C | NP_524304  NP_001097741 | Gq/11 - 0.96  Gi/o - 0.55  Gq/11 - 0.96  Gi/o - 0.62 |
| TkR99D | NP_524556  NP_001163772  NP_001263092 | Gq/11 - 0.93  Gq/11 - 0.81  Gq/11 - 0.94 |
| sNPF-R | NP_524176  NP_001262086 | Gi/o - 0.99  Gq/11 - 0.56  G12/13 - 0.38  Gi/o - 0.99  Gq/11 - 0.56  G12/13 - 0.38 |
| Dh31-R | NP_725278  NP_001260950  NP_001260951 | Gs - 0.83  Gi/o - 0.83  Gs - 0.88  Gi/o - 0.66  Gq/11 - 0.32  Gi/o - 0.94  Gs - 0.91 |
| AkhR | NP_477387  NP_723206  NP_995639  NP_001260149 | Gi/o - 0.99  Gi/o - 0.99  Gi/o - 0.99  Gi/o - 0.99 |
| Dop1R1 | NP_477007  NP_001163607  NP_001247092  NP_001262563  NP_001303454 | Gq/11 - 0.62  Gq/11 - 0.62  Gq/11 - 0.62  Gq/11 - 0.57  Gq/11 - 0.62 |
| Dop1R2 | NP_733299  NP_524548  NP_001263072 | Gq/11 - 0.99  Gq/11 - 0.99  Gq/11 - 0.98 |
| Dop2R | NP_001014759  NP_001014757  NP_001285477  NP_001014758  NP_001014760  NP_001027080 | Gi/o - 0.98  Gi/o - 0.54  Gq/11 - 0.42  Gi/o - 0.83  Gi/o - 0.83  Gi/o - 0.83  Gi/o - 0.72  Gq/11 - 0.56 |
| DopEcR | NP_647897  NP_001014560  NP_001014559 | Gi/o - 0.97  Gq/11 - 0.87  Gi/o - 0.97  Gq/11 - 0.87  Gi/o - 0.97  Gq/11 - 0.87 |

| **Receptor** | **Isoform** | **G-protein prediction scores** |
| --- | --- | --- |
| 5-HT1A | NP_725849 | Gi/o - 0.99 |
| 5-HT1B | NP_523789  NP_001163201  NP_001137708 | Gi/o - 0.64  Gi/o - 0.64  Gi/o - 0.64 |
| 5-HT2A | NP_524223  NP_730859  NP_001163505  NP_001163506  NP_001097684 | Gs - 0.92  Gq/11 - 0.91  Gq/11 - 0.91  Gs - 0.89  Gs - 0.96  Gi/o - 0.93  Gq/11 - 0.85  Gs - 0.83  Gq/11 - 0.85  Gs - 0.83 |
| 5-HT2B | NP_001262373  NP_649806  NP_001287238 | Gs - 0.89  Gs - 0.89  Gs - 0.89 |
| 5-HT7 | NP_524599  NP_001263131 | Gs - 0.77  Gi/o - 0.42  Gs - 0.77  Gi/o - 0.42 |
| Oamb | NP_524669  NP_732542  NP_001262774  NP_001303429  NP_001262775  NP_732541 | Gs - 0.40  Gq/11 - 0.86  Gq/11 - 0.82  Gq/11 - 0.86  Gs - 0.40  Gq/11 - 0.86 |
| Oct-TyrR | NP_524419  NP_001163494 | Gi/o - 0.96  Gi/o - 0.96 |
| Octalpha2R | NP_650754  NP_001262714  NP_001262715 | Gi/o - 0.99  Gi/o - 0.99  Gi/o - 0.97 |
| Octbeta1R | NP_651057  NP_001034064  NP_001262843  NP_001163690 | Gq/11 - 0.56  Gq/11 - 0.75  Gs - 0.73  Gq/11 - 0.56  Gs - 0.73  Gq/11 - 0.70 |
| Octbeta2R | NP_001034049  NP_001163596  NP_001247076  NP_001247077  NP_001247078  NP_001303505 | Gs - 0.60  Gi/o - 0.45  Gi/o - 0.62  Gs - 0.35  Gs - 0.60  Gi/o - 0.45  Gs - 0.60  Gi/o - 0.45  Gs - 0.60  Gi/o - 0.45  Gs - 0.60  Gi/o - 0.45 |
| Octbeta3R | NP_001034048  NP_650210  NP_001034043  NP_001034046 | Gi/o - 0.83  Gi/o - 0.88  Gs - 0.65  Gq/11 - 0.91  Gs - 0.98  Gi/o - 0.95  G12/13 - 0.32 |
